# The 10-m cotton maps in Xinjiang, China during 2018–2021

**DOI:** 10.1038/s41597-023-02584-3

**Published:** 2023-10-10

**Authors:** Xiaoyan Kang, Changping Huang, Jing M. Chen, Xin Lv, Jin Wang, Tao Zhong, Huihan Wang, Xianglong Fan, Yiru Ma, Xiang Yi, Ze Zhang, Lifu Zhang, Qingxi Tong

**Affiliations:** 1 National Engineering Research Center for Satellite Remote Sensing Applications, Aerospace Information Research Institute, Chinese Academy of Sciences, Beijing, 100101 China; 2 University of Chinese Academy of Sciences, Beijing, 100049 China; 3 Department of Geography and Planning, University of Toronto, Toronto, ON M5S 3G3 Canada; 4 School of Geographical Sciences, Fujian Normal University, Fuzhou, China; 5 Xinjiang Production and Construction Corps Oasis Eco-Agriculture Key Laboratory, College of Agriculture, Shihezi University, Shihezi, 832003 China

**Keywords:** Agroecology, Biogeography

## Abstract

Cotton maps (10 m) of Xinjiang (XJ_COTTON10), which is the largest cotton production region of China, were produced from 2018 to 2021 through supervised classification. A two-step mapping strategy, i.e., cropland mapping followed by cotton extraction, was employed to improve the accuracy and efficiency of cotton mapping for a large region of about 1.66 million km^2^ with high heterogeneity. Additionally, the time-series satellite data related to spectral, textural, structural, and phenological features were combined and used in a supervised random forest classifier. The cotton/non-cotton classification model achieved overall accuracies of about 95% and 90% on the test samples of the same and adjacent years, respectively. The proposed two-step cotton mapping strategy proved promising and effective in producing multi-year and consistent cotton maps. XJ_COTTON10 agreed well with the statistical areas of cotton at the county level (R^2^ = 0.84–0.94). This is the first cotton mapping for the entire Xinjiang at 10-meter resolution, which can provide a basis for high-precision cotton monitoring and policymaking in China.

## Background & Summary

Cotton is one of the most important natural fibre and oil crops in the world^1–3^, and it can help to reduce extreme poverty (SDG 1, Sustainable Development Goal 1) and achieve the Zero Hunger Goal (SDG 2), as part of the 2030 Agenda for SDGs^4^. According to the Organization for Economic Co-operation and Development (OECD) and the Food and Agriculture Organization (FAO) of the United Nations, Xinjiang, as the leading and largest cotton production region in China, has accounted for over 80% and about 20% of cotton production in China and the world, respectively, during these years^5,6^. The accurate and detailed mapping of cotton in Xinjiang is vital for cotton management, disease prevention and control, and yield forecast. Xinjiang covers about one-sixth of China’s total land area, with an area of more than 1.66 million square kilometers (km^2^) and is characterized by typical heterogeneous desert-oasis ecotones (sourced from http://english.www.gov.cn/archive/ (last access: June 3, 2023)). It is a challenge to produce a fine cultivation map of cotton throughout Xinjiang^7^, probably because such a task needs a powerful platform with high storage and computing capabilities^8^; it needs enough high-quality and evenly distributed samples due to the high spatial heterogeneity^9^; and it needs models with high universality, accuracy, and transferability to address issues like bad weather^10^. To date, numerous studies have mainly focused on Xinjiang cotton mapping at county^1,11,12^, prefecture^13^, region^7,14^, and province scales^15,16^. Although we recently have produced the 500 m nationwide cotton maps in 2016 and 2018 that were included in the reports on remote sensing monitoring of China Sustainable Development^15,16^, both of them demonstrated limitations and disadvantages in practical precision management and decision making due to the very low spatial resolutions. To our knowledge, there were currently no fine-resolution large-scale cotton maps covering the entire Xinjiang.

It is of great importance to choose a suitable computing platform and remote sensing data for cotton mapping across Xinjiang, for satellite-based remote sensing imagery has been developed as the main data source for crop mapping in large spatial ranges^17,18^. With such data, a variety of crop mapping datasets have been produced and released successively, such as the Cropland Data Layer (CDL) of the United States^19^, the Agriculture and Agri-Food Canada’s Annual Crop Inventory (AAFC)^20^, the Early-Season Mapping of Winter Wheat in China (EMWC)^21^, and the Crop Mapping in Northeast China (CMNEC)^22^. Among them, the imagery sources mainly came from Landsat series^21,23^, SPOT series^20^, and Sentinel series^22^. Besides these large series datasets, GaoFen series and PlanetScope are another two datasets for the fine classification of crops^24–27^. However, the crop mapping for a vast area of a million km^2^ often needs to handle images with tens of thousands of scenes^28^, which are beyond the capacity of local workstations. The developing platforms with the capabilities of cloud storage and computing, such as the Google Earth Engine (GEE), have significantly aided the advancement of crop mapping at regional and global scales^29,30^. Ten years after the launch of GEE (2010–2019), more than 70 studies have been conducted in crop mapping using such a platform^31^.

Optical and radar remote sensing imagery are two main types of satellite data, both of which can provide abundant phenological and spatial information with spectral reflectance and backscattering coefficient, respectively^18^. Because of phenological differences among various crops, phenological characteristics and curves are often used to identify crops^13,17,32^. Spatial features such as the Gray-Level Co-occurrence Matrix (GLCM) indices have proven effective for improving the performance of crop classification^33,34^. As an active remote sensing technology, radar can effectively depict the spatial structure of land cover in cloudy and rainy weather, which is hard for optical remote sensing^35^. Due to their complementarity, the combination of optical and radar remote sensing has been a widely used strategy in crop classification^1,7,28^. In addition, there are two types of common auxiliary data, i.e., topographic and meteorological variables^19,36^. Topographic variables such as digital elevation model (DEM) and slope can aid in the identification of irrigated cropland^37^ and terraced fields^38^. Meteorological variables such as temperature and rainfall greatly affect plant growth and development and hence have strong correspondence with plant phenology^39^. Therefore, the combination of optical and radar remote sensing imageries as well as auxiliary data may offer more information gains for crop classification across Xinjiang.

In this study, we adopted all available Sentinel-1 and 2 imageries as well as auxiliary data on the GEE platform to develop 10 m cotton maps in Xinjiang from 2018 to 2021. Due to different availability of images in different years, we designed differentiated approaches and carried out detailed comparison and evaluation. The proposed method can be transferred to cotton mapping in a new year, and the published cotton maps dataset can provide a basis for crop management and policymaking.

## Methods

### Study area

**Fig. 1:**
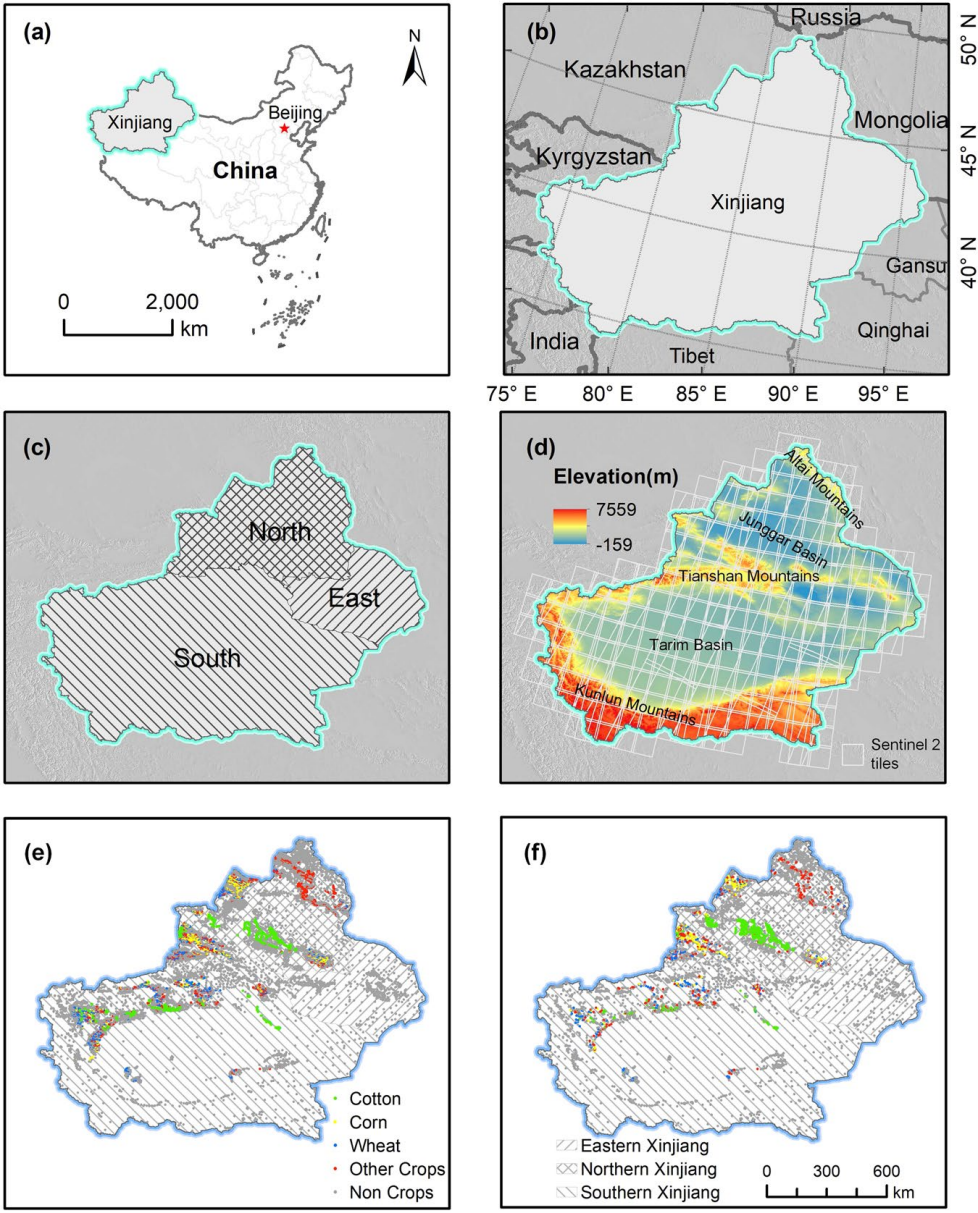
The location (**a,b**), geographical zones (**c**), topographic condition (**d**), and ground truth samples (2020 (**e**) and 2021 (**f**)) of Xinjiang, China. The administrative boundary data of global countries and provinces in China are from http://www.resdc.cn/ (last access: June 3, 2023) © Institute of Geographic Sciences and Natural Resources Research, Chinese Academy of Sciences.

Xinjiang is located in Northwest China (73°46′ ~ 96°23′E; 34°25′ ~ 49°50′N)^40^ (Fig. 1a,b), covering an area of about 1.66 million km^2^. It is the largest provincial administrative region in China, accounting for one-sixth of China’s total land area and with a land border of about 5600 kilometers (Fig. 1b). The terrain of Xinjiang is characterized by two basins (the Junggar Basin and Tarim Basin) between three mountains (Altay, Tianshan, and Kunlun Mountains) from north to south, and it is typically divided into three parts by the middle mountain Tianshan and climate zones, i.e., Northern Xinjiang (NX), Southern Xinjiang (SX), and Eastern Xinjiang (EX) (Fig. 1c,d). According to the Yearbooks of Xinjiang^40,41^, the main crops in Xinjiang are wheat, corn, and cotton, of which cotton is the largest crop in recent five years.

### Data sets

According to the Yearbooks in Xinjiang^42,43^, we carried out field sampling in the main-cotton-planting areas in 2020 and 2021, respectively. A large number of positioning points for crops (including cotton, wheat, corn, and other crops) and non-crop (developed land, water, forest, grass, desert, mountain, etc.) were collected (Table 1). Further, to obtain enough high-quality and evenly distributed training data across the entire Xinjiang, sample expansion was performed by visual interpretation of very high-resolution imagery from the Google Earth platform^7,13,14^. As a result, a total of 10418 samples for 2020 (2627 samples for non-cotton crops and 7791 samples for non-crop) and a total of 5422 samples for 2021 (1147 samples for non-cotton crops and 4275 samples for non-crop) were acquired (Table 1). The spatial distributions of all samples in 2020 and 2021 are presented in Fig. 1e,f.

**Table 1:** The total numbers of samples from different sources for each crop.

Class name	2020			2021		
	Field samples	Google Earth samples	The total	Field samples	Google Earth samples	The total
Cotton	3082	0	3082	2311	0	2311
Corn	466	598	1064	0	290	290
Wheat	154	699	853	0	254	254
Other crops	341	1330	1671	0	603	603
Non-crop	1018	7791	8809	0	4275	4275

#### Surface reflectance

The Sentinel series imagery covering optical (Sentinel 2 A/B, S2) and radar (Sentinel 1, S1) imagery provides multi-band images with a spatial resolution of 10 m for Xinjiang from 2018 to 2021. S2 bottom-of-atmosphere (BOA) reflectance imagery from 2019 to 2021 and top-of-atmosphere (TOA) reflectance imagery from 2018 to 2021 are available in the GEE platform for Xinjiang. In general, S2 BOA reflectance imagery collection was often used to classify crops^21,44,45^. As shown in Fig. 1d,there are about 230 tiles in this study area, with a total of 31,945 scenes in 2018 (Table 2). Given the massive computing and storage demands, we did not pre-process the TOA images to BOA images. Instead, we used TOA imagery for cotton mapping in 2018 based on You *et al*.^22^ and You and Dong^28^.

**Table 2:** Summary of S2 imagery in Xinjiang.

Year	Scenes		Bands	Resolution
	BOA	TOA		
2018	—	31,945	B2 (Blue), B3 (Green), B4 (Red), B8 (NIR)	10 m
2019	33,628	33,628		
2020	33,593	33,593		
2021	33,379	33,379		

To remove clouds and cloud shadows from TOA/BOA images, we adopted the following steps to process S2 data^46^:

1. obtaining clean pixels: employing the GEE-provided cloud probability dataset to generate clean pixels for each TOA/BOA image^47,48^.
2. Monthly synthesizing surface reflectance (SR): based on clean pixels, using the monthly median value as the SR value^28^.
3. Smoothing time-series data: eradicating null values and extreme values by deploying the Savitzky-Golay filter with a fitting window of 9 and a polynomial degree of 2^22^.

#### Backscattering coefficient

Previous studies have reported that radar backscatter coefficients could capture crop structure, ground conditions, and their interaction information^10,49^. Therefore, S1 C-band (5.405 GHz) synthetic aperture radar (SAR) imagery collection was also included in this study (Table 3). The GEE platform provides the ground range detected (GRD) imagery with two bands of the interferometric wide (IW) swath mode, i.e., single co-polarization VV (vertical transmit/vertical receive) and dual-band cross-polarization VH (vertical transmit/horizontal receive). The provided imagery was pre-processed with the S1 Toolbox, sequentially by thermal noise removal, radiometric calibration, basic terrain correction for areas greater than 60° latitude, and finally scaling (10*log10(x)) as dB^10,50^. Besides, we further processed the GRD imagery with VV and VH bands by additional border noise correction, speckle filtering, and radiometric terrain normalization, according to Tian *et al*.^10^ and Mullissa *et al*.^50^ Additionally, similar to the S2 data, we performed denoising on the S1 data by the Savitzky-Golay filter, with a fitting window of 7 and a polynomial degree of 2.

**Table 3:** Summary of S1 imagery in Xinjiang.

Year	Scenes	Bands	Resolution
2018	4,578	VV, VH	10 m
2019	4,106		
2020	4,367		
2021	4,289		

#### Topographic variables

As shown in Fig. 1d, we employed the 30 m shuttle radar topography mission (SRTM) digital elevation model (DEM) data^51^ in this study. Besides DEM, the GEE platform provides its slope and aspect layers, which are often used in land cover change detection and classification^37,38,52^.

#### Meteorological variables

Previous studies have shown that temperature and rainfall exhibit strong correspondence with cropping patterns and phenological metrics in agricultural areas^36,37,39^. Annual mean temperature and precipitation from WorldClim Version 1^53^ were employed here as two of the auxiliary datasets.

#### Geographical zones

In general, Xinjiang consists of NX, EX, and SX, as shown in Fig. 1c. These regions have different planting structures for major crops and different meteorological environments^7,14,54^. Referring to You *et al*.^22^, independent experiments and analyses were carried out in each geographical zone to eliminate spatial heterogeneity of cotton phenology.

#### Yearbooks

The statistical data in the yearbook is often used to evaluate the crop mapping performance^21,22^. There are two series of yearbooks in Xinjiang, i.e., the Yearbooks of Xinjiang Uygur Autonomous Region (hereinafter, Xinjiang(Local) for short) and the Yearbooks of Xinjiang Production and Construction Corps (hereinafter, Xinjiang(Corps) for short). They are independently compiled by the Statistic Bureaus of Xinjiang(Local) and Xinjiang(Corps), respectively. There are a total of 94 counties (including county-level cities) from Xinjiang(Local) (85 counties) and Xinjiang(Corps) (9 counties). Up to now, the annual planting areas from 2018 to 2020 have been published through the two series of yearbooks^40,41,43,55,56^.

### Feature selection

In order to avoid overfitting and improve generalization ability, we preferred multi-sensor features with high independence considering spectral, textural and structural properties, respectively. We hence chose EVI (enhanced vegetation index, Eq. (1)) from S2^22,28^, SAVG (sum average, Eq. (2)) of the blue band from S2^34,57^, and the VH band from S1^23,28^ as the main independent variables.

1 $$EVI=2.5\times \frac{{\rm{NIR}}-{\rm{Red}}}{{\rm{NIR}}+6\times {\rm{Red}}-7.5\times {\rm{Blue}}+1},$$

2 $$SAVG={\sum }_{k=2}^{2{N}_{g}}k\left({\sum }_{i=1}^{{N}_{g}}{\sum }_{j=1}^{{N}_{g}}p\left(i,j\right)\right),i+j=k,$$

where *p*(*i,j*) is the (*i,j*)th entry in the GLCM of a pixel; *N*_*g*_ is the number of distinct gray levels in the quantized image^58^. In each GLCM, the size of the neighborhood was set to 4, and the kernel was set to a 9 × 9 square (radius = 4 pixels).

Besides, topographic and meteorological factors are important in distinguishing croplands^37,38,52^. Therefore, we employed three topographic variables and two meteorological variables, i.e., DEM, Slope, Aspect, temperature (Temp), and precipitation (Prec).

**Fig. 2:**
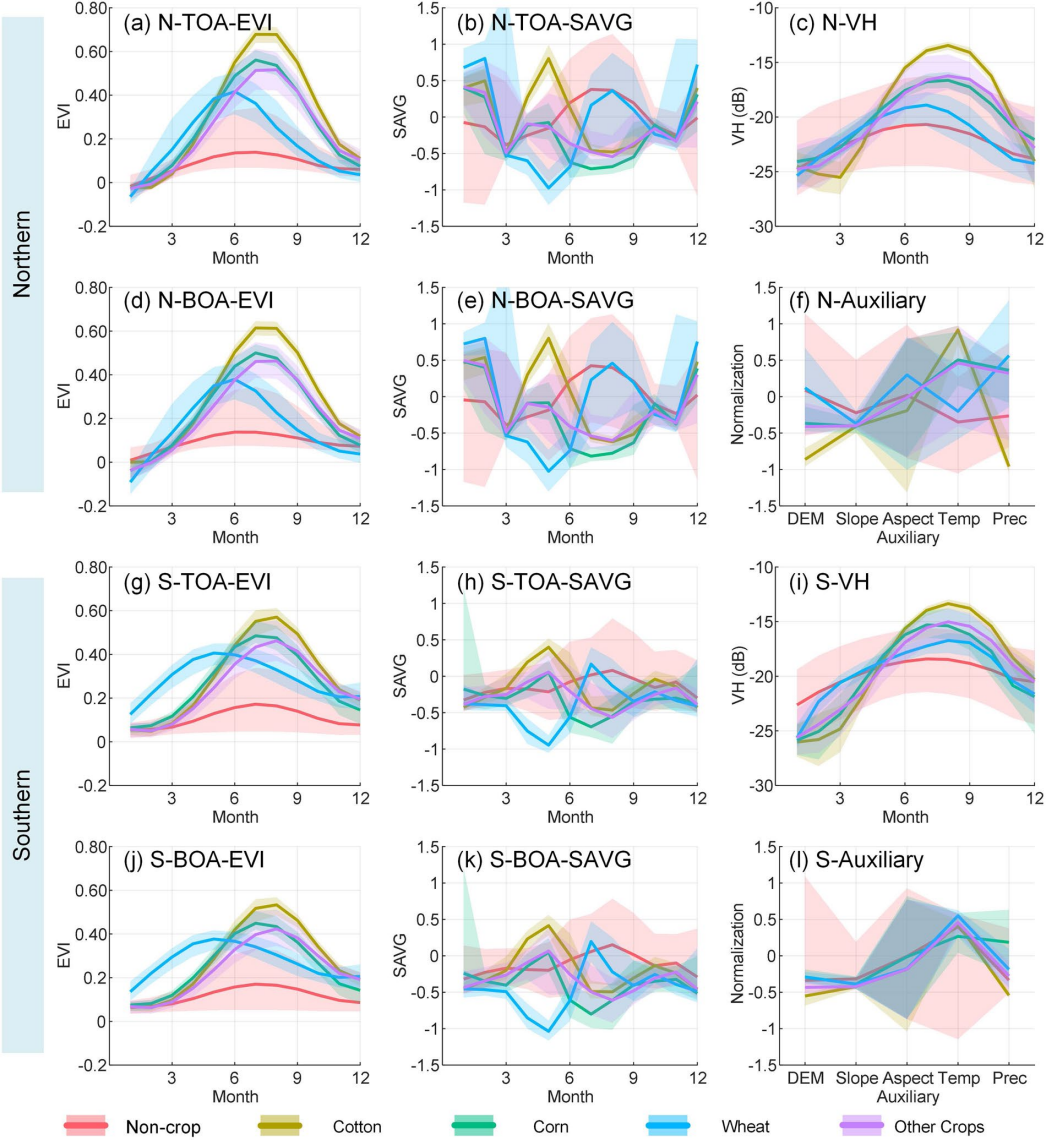
Potential analysis of various features in crop classification. The summary curves display the 25%, 50% (median), and 75% percentiles of a feature for each class. For better presentation, the SAVG and Auxiliary features were normalized by centering with mean 0 and scaling by standard deviation 1.

In terms of phenological curves between SX and NX, different crops and non-crop presented varied differences, according to Fig. 2. First, the EVI curves presented more obvious differences between crops and non-crop in NX than in SX. The maximum EVI values of wheat appeared in different months in SX (May) and NX (June). The EVI values of cotton from June to September were higher in NX than in SX. Second, the SAVG feature from December to February expressed obvious differences between crops and non-crop in NX but not in SX. Third, the VH curves indicated the stronger separability between cotton and non-cotton crops in NX than in SX. Moreover, the auxiliary features revealed that crops and non-crop were more separable in NX than in SX, especially Slope, Temp, and Prec. These details hence suggested the necessity of the zoning mapping strategy.

According to recent studies in crop classification and mapping^7,10,21,22^, by employing a two-step strategy, we planned to distinguish cropland from other lands and then performed the cotton/non-cotton classification within the cropland to map cotton. The two-step strategy can eliminate interference from non-cropland types and thus make cotton mapping more convenient and accurate. As seen in Fig. 2, some features, such as EVI and VH (from June to September), had great potential to distinguish cotton and non-cotton types, but may misclassify wheat fields and non-crop lands. By contrast, the EVI features from April to June had high potential for distinguishing cropland from non-crop lands but can hardly be used to classify crops, especially in NX. These preliminary results revealed the importance of the two-step mapping approach and the necessity of a classifier that can effectively select suitable features from the feature set.

Besides, there were obvious similarities in outstanding features in distinguishing cropland and cotton. For example, the SAVG feature in May can distinguish cotton from non-cotton and non-crop lands in both SX and NX. Notably, the feature curves derived from TOA imagery were quite similar to those derived from BOA imagery, indicating that TOA imagery can be used for crop mapping in this study.

### Cotton mapping strategy

Given that a classification model in one spacetime can be transferred to another similar one^59^ and that crops in adjacent years and regions have similar characteristics, including phenological, topographic, and meteorological variables, transfer learning was often used in crop classification in Xinjiang^11,12,15,16^. As illustrated in Fig. 1e,f and Table 1, there were enough samples in 2020 and 2021 but none in 2018–2019. Meanwhile, we did not perform field sampling in EX because it is not a main-cotton-planting area. Therefore, transfer learning was employed: (1) transferring the models trained on the samples in 2020 to distinguish cropland and cotton in 2018–2019; and (2) transferring the models trained on the samples in NX to distinguish cropland and cotton in EX.

As shown in Table 2, the GEE platform provides no BOA imagery for this study area in 2018. Therefore, we designed two combinations about the selected features: (1) BOA^+^: time-series EVI and SAVG derived from the S2 BOA imagery, time-series VH, and the auxiliary features; and (2) TOA^+^: time-series EVI and SAVG derived from the S2 TOA imagery, time-series VH, and the auxiliary features.

**Fig. 3:**
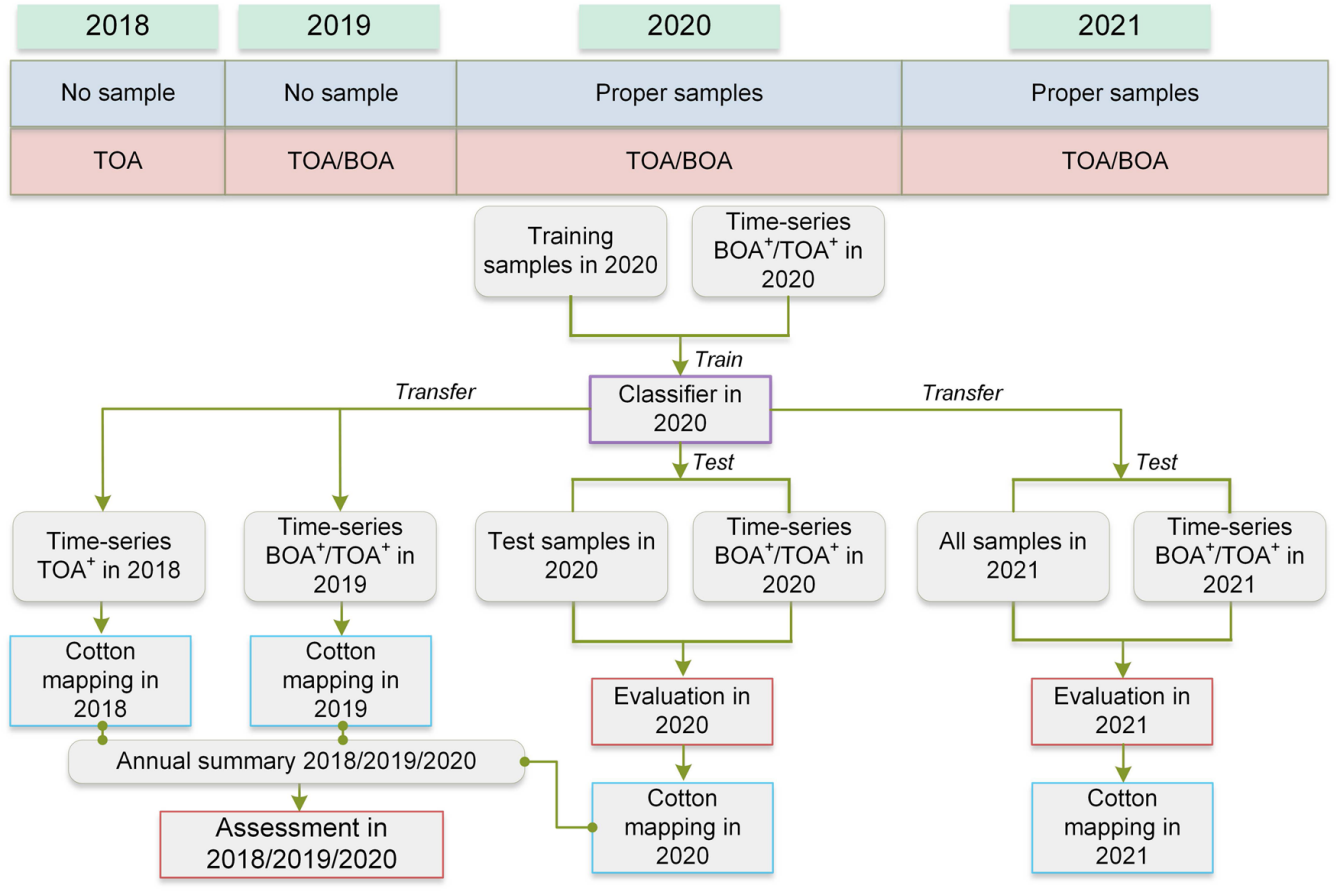
The framework of cotton mapping of Xinjiang during 2018–2021.

The GEE platform provides multiple machine learning methods for imagery classification, such as random forest (RF), decision tree, support vector machine, and naive bayes classifiers. Among them, RF is commonly used in crop classification^22,23,28,38^. Because the RF classifier can employ the bagging technique to seek optimal features via the auto optimization mechanism for better classification^60^, we performed the models’ training with the same feature set for the cropland mapping and the cotton mapping. Taking the second step of cotton mapping as an example, the technical framework was provided in Fig. 3. It mainly included the training and test of the classification model in 2020 (Evaluation in 2020), the performance evaluation of the transfer ability in 2021 (Evaluation in 2021), and cotton planting area assessment in 2018–2020 (Assessment in 2018/2019/2020). Finally, we can obtain the 10-m cotton maps during 2018–2021 via the TOA^+^ approach and three 10-m cotton maps during 2019–2021 via the BOA^+^ approach.

For evaluating the models in 2020 and 2021, we adopted the following five metrics: OA (overall accuracy), Kappa (Kappa coefficient), UA (user’s accuracy), PA (producer’s accuracy), and the F1-score^28^. On the assessment of cotton planting areas between the estimation and the summary, we employed R^2^ (the coefficient of determination), *p* value (the significance level), and RMSE (the root mean square error)^21^.

### Postprocessing

The salt and pepper noise often appears in the classification result of a vast area, mainly because of different objects with the same spectrum or the same objects with different spectra^38,61^. To address this issue, edge-preserving filtering with a bilateral filter^62^ was employed. For the used bilateral filter, the standard deviation of the spatial Gaussian smoothing kernel was set to 1, and the size of the square neighborhood around each pixel was set to 5 pixels^63^.

## Data Records

The XJ_COTTON10 dataset^64^ (10-m cotton maps of Xinjiang from 2018 to 2021) is available to the public at 10.5281/zenodo.7856467. It includes the following seven compressed packages of 30 geofiles with a size of 3° × 3°:

1. Cotton_2018_TOA_10m_filter.zip: the 10 m cotton map in 2018 via the TOA^+^ approach;
2. Cotton_2019_BOA_10m_filter.zip: the 10 m cotton map in 2019 via the BOA^+^ approach;
3. Cotton_2019_TOA_10m_filter.zip: the 10 m cotton map in 2019 via the TOA^+^ approach;
4. Cotton_2020_BOA_10m_filter.zip: the 10 m cotton map in 2020 via the BOA^+^ approach;
5. Cotton_2020_TOA_10m_filter.zip: the 10 m cotton map in 2020 via the TOA^+^ approach;
6. Cotton_2021_BOA_10m_filter.zip: the 10 m cotton map in 2021 via the BOA^+^ approach;
7. Cotton_2021_TOA_10m_filter.zip: the 10 m cotton map in 2021 via the TOA^+^ approach.

The geographic reference (ESPG: 4326 (WGS_1984)) is the same for all seven maps, conforming to that in the geotiff file. The values of the seven maps contains 1 and 0, representing cotton and other land (including other crops and non-cropland).

## Technical Validation

### Model evaluation

During the RF classifier training and validation, the randomly selected half of the total samples for each type were used as the training samples, and the rest were used as the test samples, to ensure enough samples in both training and test^22,65^. The number of trees was set to 100, and the number of variables per split was set as the square root of the number of variables (41)^22^. The estimated labels of the test samples can be achieved by a corresponding classification model for each zone (Fig. 1c). We combined the estimated labels and ground truth labels of all zones together to produce a total confusion matrix. Table 4 illustrates the summary of the accuracy assessment of crop/non-crop classification models on samples in 2020 and 2021. The crop mapping models of the two strategies (testing by the samples in the same year and the adjacent year) had OAs of over 95% and F1-scores of cropland of over 95%, indicating that transfer learning was feasible on crop mapping across Xinjiang. Besides, the two feature-combinations of TOA^+^ and BOA^+^ had close performance on the five statistical indices, suggesting that S2 TOA imagery worked well on cropland mapping in this study area.

**Table 4:** Performance evaluation of crop/non-crop classification models.

Strategy	Features	Class	Non-crop	Crop	PA (%)	UA (%)	F1-score (%)
M20	TOA^+^	Non-crop	4250	154	96.50	97.03	96.77
		Crop	130	3203	96.10	95.41	95.75
		OA = 96.33%			Kappa = 0.9252		
	BOA^+^	Non-crop	4252	152	96.55	96.75	96.65
		Crop	143	3190	95.71	95.45	95.58
		OA = 96.19%			Kappa = 0.9223		
M21	TOA^+^	Non-crop	2057	80	96.26	96.57	96.41
		Crop	73	1654	95.77	95.39	95.58
		OA = 96.04%			Kappa = 0.9199		
	BOA^+^	Non-crop	2057	80	96.26	96.35	96.30
		Crop	78	1649	95.48	95.37	95.43
		OA = 95.91%			Kappa = 0.9173		
M20T21	TOA^+^	Non-crop	4062	213	95.02	97.18	96.09
		Crop	118	3340	96.59	94.01	95.28
		OA = 95.72%			Kappa = 0.9137		
	BOA^+^	Non-crop	4066	209	95.11	96.86	95.98
		Crop	132	3326	96.18	94.09	95.12
		OA = 95.59%			Kappa = 0.9110		

M20 denotes the strategy of training a model on half of the total samples in 2020 and testing it on the other half. M21 denotes the strategy of training a model on half of the total samples in 2021 and testing it on the other half. M20T21 refers to the strategy of training a model on half of the total samples in 2020 and testing it on all samples in 2021.

According to the summary of the accuracy assessment of cotton/non-cotton classification models on samples in 2020 and 2021 (Table 5), The strategy (M20 and M21) of testing by the samples in the same year achieved OAs of over 94% and F1-scores of cotton of over 95%, which were about 4–5% and about 3% higher than the transfer learning strategy M20T21, respectively. Despite all this, the transfer learning strategy, which had an OA of over 90% and an F1-score of cotton of over 92%, can be considered for cotton mapping in adjacent years without samples. Moreover, S2 TOA imagery also worked well on cotton mapping, considering the small performance differences between the two feature-combinations of TOA^+^ and BOA^+^.

**Table 5:** Performance evaluation of cotton/non-cotton classification models.

Strategy	Features	Class	Non-cotton	Cotton	PA (%)	UA (%)	F1-score (%)
M20	TOA^+^	Non-cotton	1721	71	96.04	96.09	96.06
		Cotton	70	1471	95.46	95.40	95.43
		OA = 95.77%			Kappa = 0.9149		
	BOA^+^	Non-cotton	1708	84	95.31	96.17	95.74
		Cotton	68	1473	95.59	94.61	95.09
		OA = 95.44%			Kappa = 0.9083		
M21	TOA^+^	Non-cotton	519	53	90.73	92.68	91.70
		Cotton	41	1114	96.45	95.46	95.95
		OA = 94.56%			Kappa = 0.8765		
	BOA^+^	Non-cotton	518	54	90.56	92.01	91.28
		Cotton	45	1110	96.10	95.36	95.73
		OA = 94.27%			Kappa = 0.8701		
M20T21	TOA^+^	Non-cotton	1052	95	91.72	81.55	86.34
		Cotton	238	2073	89.70	95.62	92.57
		OA = 90.37%			Kappa = 0.7894		
	BOA^+^	Non-cotton	1055	92	91.98	81.47	86.40
		Cotton	240	2071	89.61	95.75	92.58
		OA = 90.40%			Kappa = 0.7903		

M20 denotes the strategy of training a model on half of the total samples in 2020 and testing it on the other half. M21 denotes the strategy of training a model on half of the total samples in 2021 and testing it on the other half. M20T21 refers to the strategy of training a model on half of the total samples in 2020 and testing it on all samples in 2021.

### Area assessment

**Fig. 4:**
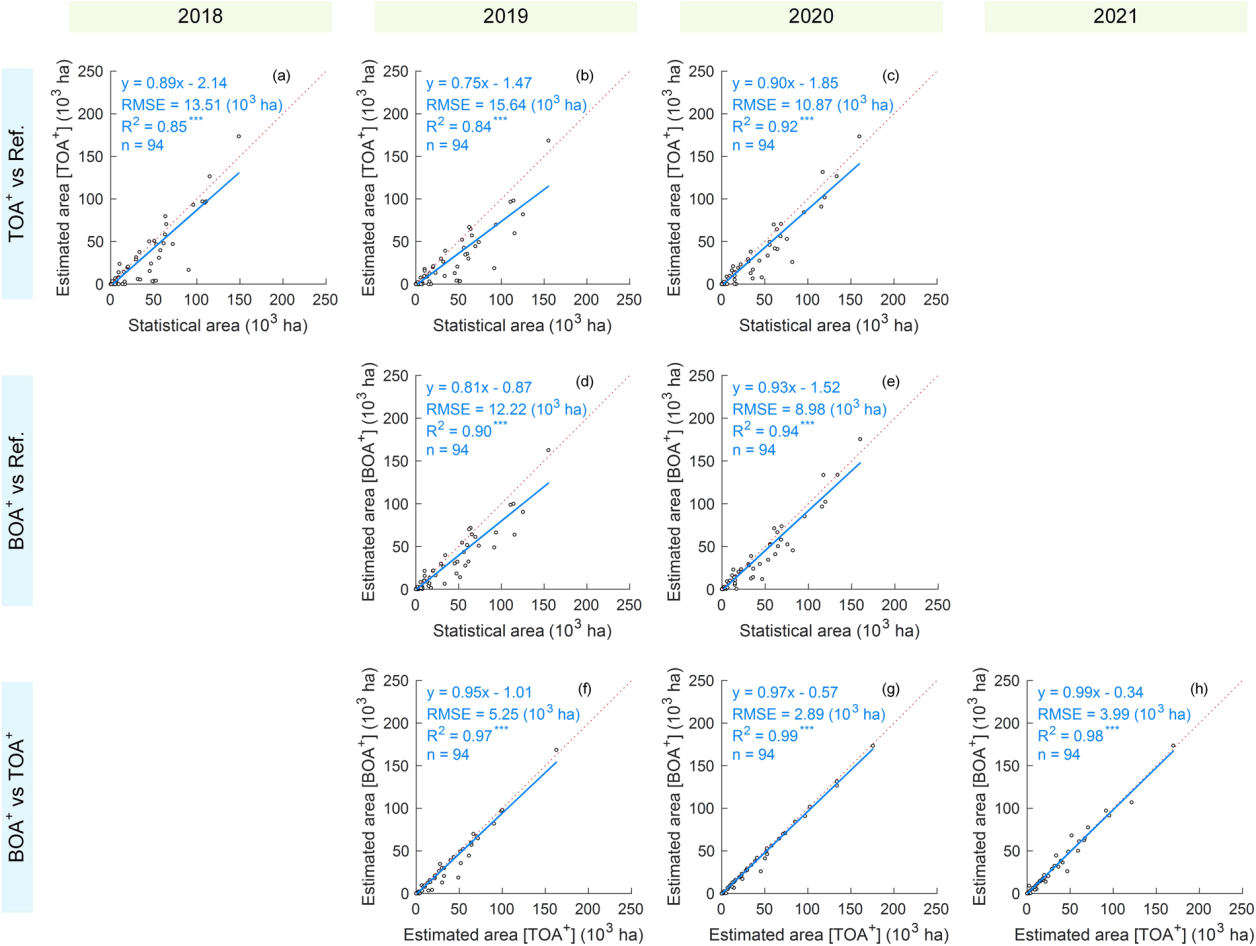
Comparison among the estimated plant areas by TOA^+^ and BOA^+^ and the agricultural statistical area (Ref.) at the county level. The red dotted line denotes the 1:1 line. Significance level: ***(p < 0.001).

Using the cotton mapping strategies, cotton maps during 2018–2021 via the TOA^+^ and BOA^+^ approaches were produced on the GEE platform. Notably, cropland and cotton maps in 2018, 2019, and 2021 were developed by the transfer learning strategy of the models trained on the samples in 2020. We further computed the total cotton-planting area at the county level for the seven cotton maps on the GEE platform. Combining the estimated areas by cotton maps and the statistical areas by the two series of yearbooks in Xinjiang, the performance evaluation of the cotton mapping was delivered in Fig. 4. Results showed that our maps accurately estimated the areas of cotton at the county level, with R^2^ values ranging from 0.84 to 0.94. By contrast, the transfer learning strategy based on BOA^+^ (R^2^ = 90 in 2019) provided slightly better performance than TOA^+^ (R^2^ = 0.84–0.85 in 2019 and 2018). Moreover, from the comparisons between TOA^+^ and BOA^+^ (Fig. 4f–h), there were slight differences in county-level cotton-planting area estimations between them, suggesting the feasibility of the TOA^+^ strategy.

### Uncertainty analysis

**Fig. 5:**
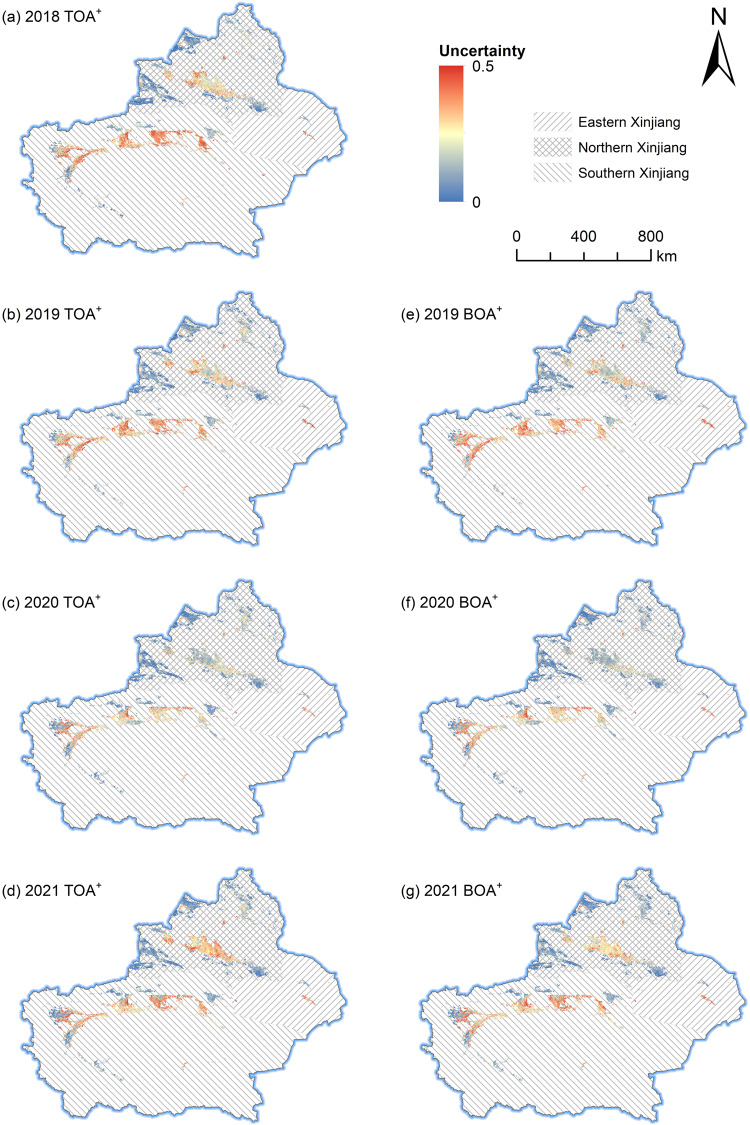
Pixel-level uncertainty of cotton mapping from 2018 to 2021. The maps were resampled to 1 km × 1 km spatial resolution for clearer visual effect. The administrative boundary file comes from http://www.resdc.cn/ (last access: June 3, 2023) © Institute of Geographic Sciences and Natural Resources Research, Chinese Academy of Sciences.

**Fig. 6:**
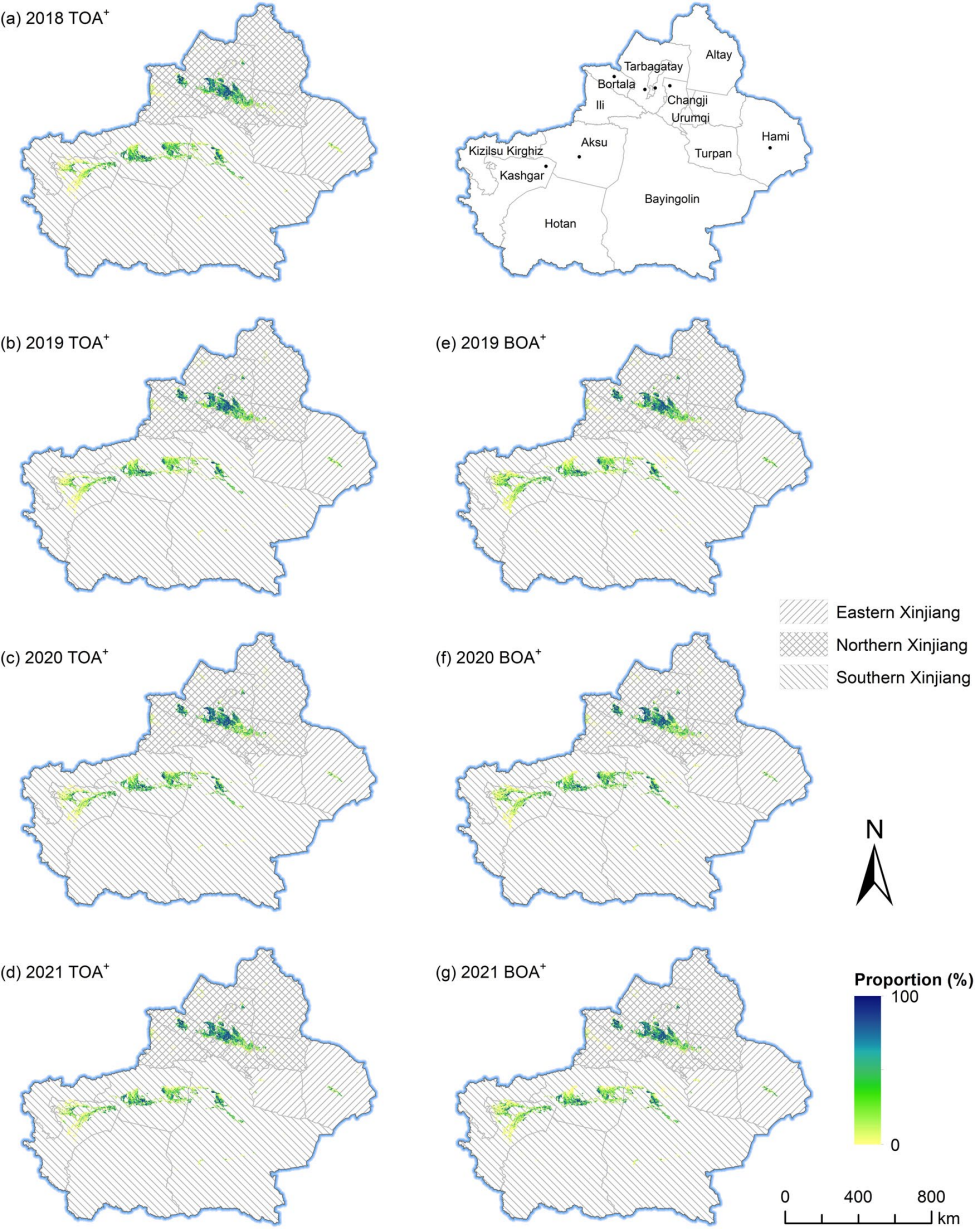
Cotton distribution across Xinjiang from 2018 to 2021. The map presents the proportion of cotton within each 1 km × 1 km grid cell. The seven typical cotton-planting areas shown in Fig. 7 are marked as black points. The administrative boundary file comes from http://www.resdc.cn/ (last access: June 3, 2023) © Institute of Geographic Sciences and Natural Resources Research, Chinese Academy of Sciences.

We adopted the classification probability of each pixel in cropland maps (cotton/non-cotton) to generate its uncertainty by 1-*P*_max_, where *P*_max_ was the maximum probability of being classified as cotton/non-cotton types^38,66^. As seen in Fig. 5, by contrast, the uncertainties of crop pixels were lower in NX than in SX overall. Cropland fragmentation may be a main reason. In NX, there is a large amount of farmland that has been integrated into contiguous areas by Xinjiang(Corps) since the 1950s^67,68^. However, in SX, the majority of croplands were managed by individual farmers, resulting in fragmented lands with diverse crops^67^. As shown in Fig. 6, the cotton proportion maps at the kilometer scale revealed that there were large areas of concentrated and contiguous high-standard farmland for cotton planting in NX but large areas of dispersed cotton in SX. Previous studies have indicated that cropland fragmentation and heterogenization can make farmland management inefficient^69^ and crop yields decrease^70–72^. Such a land use pattern can lead to a large number of mixed pixels and decrease the representativeness of field samples, leading to uncertainty in classification^73^.

**Fig. 7:**
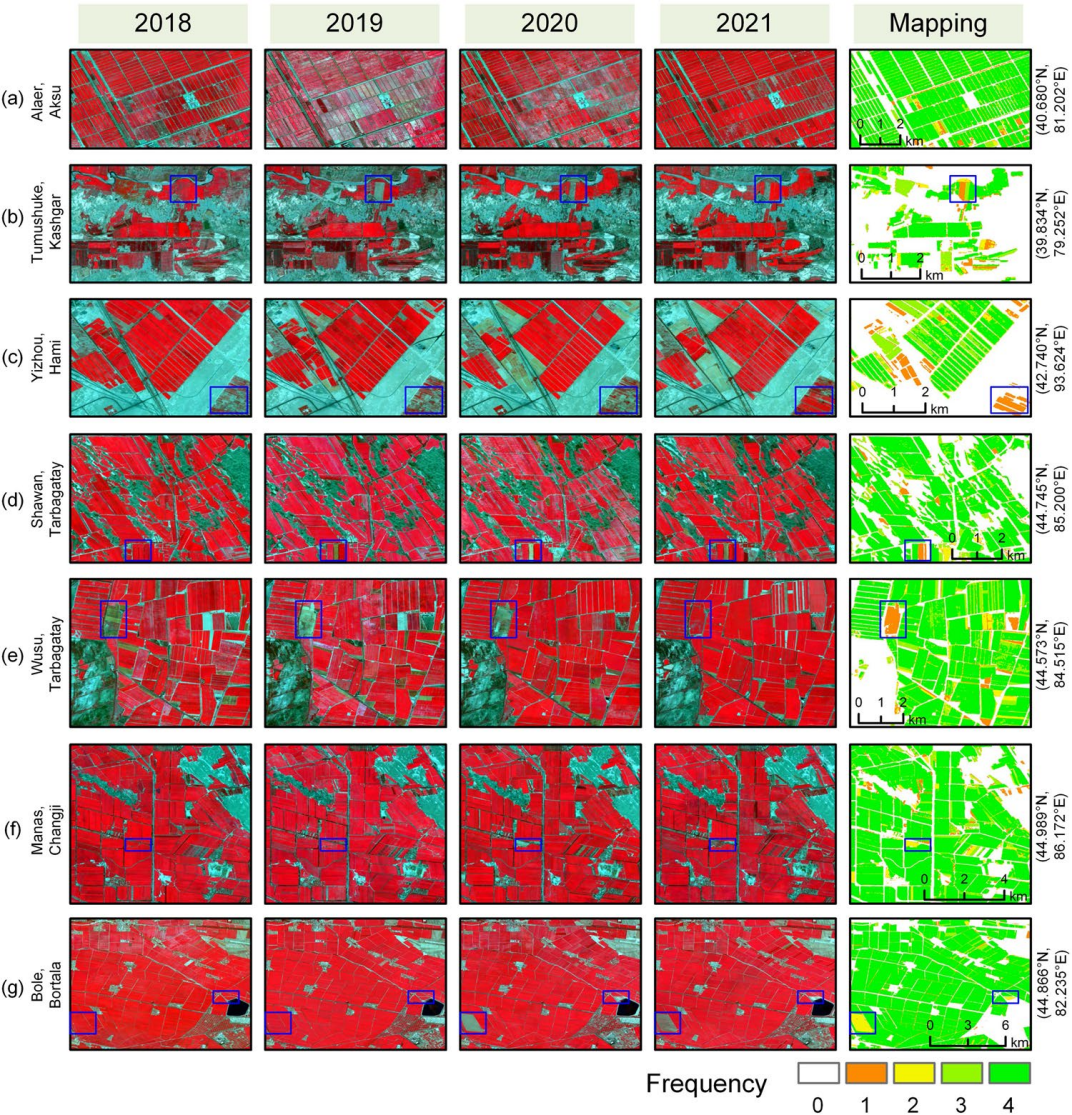
Selected case comparison of Sentinel-2 imagery (NIR-Red-Green, median composite from 1 May to 1 September) and cotton mapping results from 2018 to 2021 at seven different locations (**a**–**g**). Among them, (**a,b**) are in Southern Xinjiang, (**c**) is in Eastern Xinjiang, and (**d**–**g**) are in Northern Xinjiang. The geographic coordinates express the centers of the selected cases. The blue boxes denote cotton with typical dynamics.

According to cotton distribution during 2018–2021, cotton is mainly in SX and NX (Fig. 6). At the prefecture level, the most densely distributed areas were in Bortala, Tarbagatay, Changji, Aksu, and Bayingolin. By contrast, cotton-planting fields were dispersed overall in SX but more concentrated in NX. We selected seven typical cotton-planting cases, which are in Alaer (Aksu), Tumushuke (Kashgar), Yizhou (Hami), Shawan (Tarbagatay), Wusu (Tarbagatay), Manas (Changji), and Bole (Bortala), and detailed their cotton dynamics during the last four years (Fig. 7). Visually, our cotton maps realistically provided most of the distributions and the dynamics of cotton. The blue boxes marked in Fig. 7b–g displayed a few cotton parcels with typical dynamic changes, and our maps clearly captured the details. For example, the displayed cotton-planting frequency in the blue boxes in Fig. 7e was in line with the dynamic of the field in these four years. The dynamics of cotton like these were generally accurately captured in our maps. Apart from the misclassified pixels near the edge of field parcels due to the mixed pixel issue, a number of roads were clearly seen between the parcels, highlighting the advantage of 10-m field maps over coarse maps^15,16^.

Transfer learning in crop classification is based on the basic postulate that crops in adjacent years and regions have similar characteristics including phenological, topographic and meteorological variables. However, as the interval increases, the similarity of the same crop in different years may lessen. Overall, the uncertainties in 2020 were lower than those in the other three years (Fig. 5).

To tackle the two problems mentioned above, adding more independent samples with high representativeness, especially in SX, and estimating cotton distributions at the subpixel level are two routes worth considering in future work.

### Feature importance evaluation

**Fig. 8:**
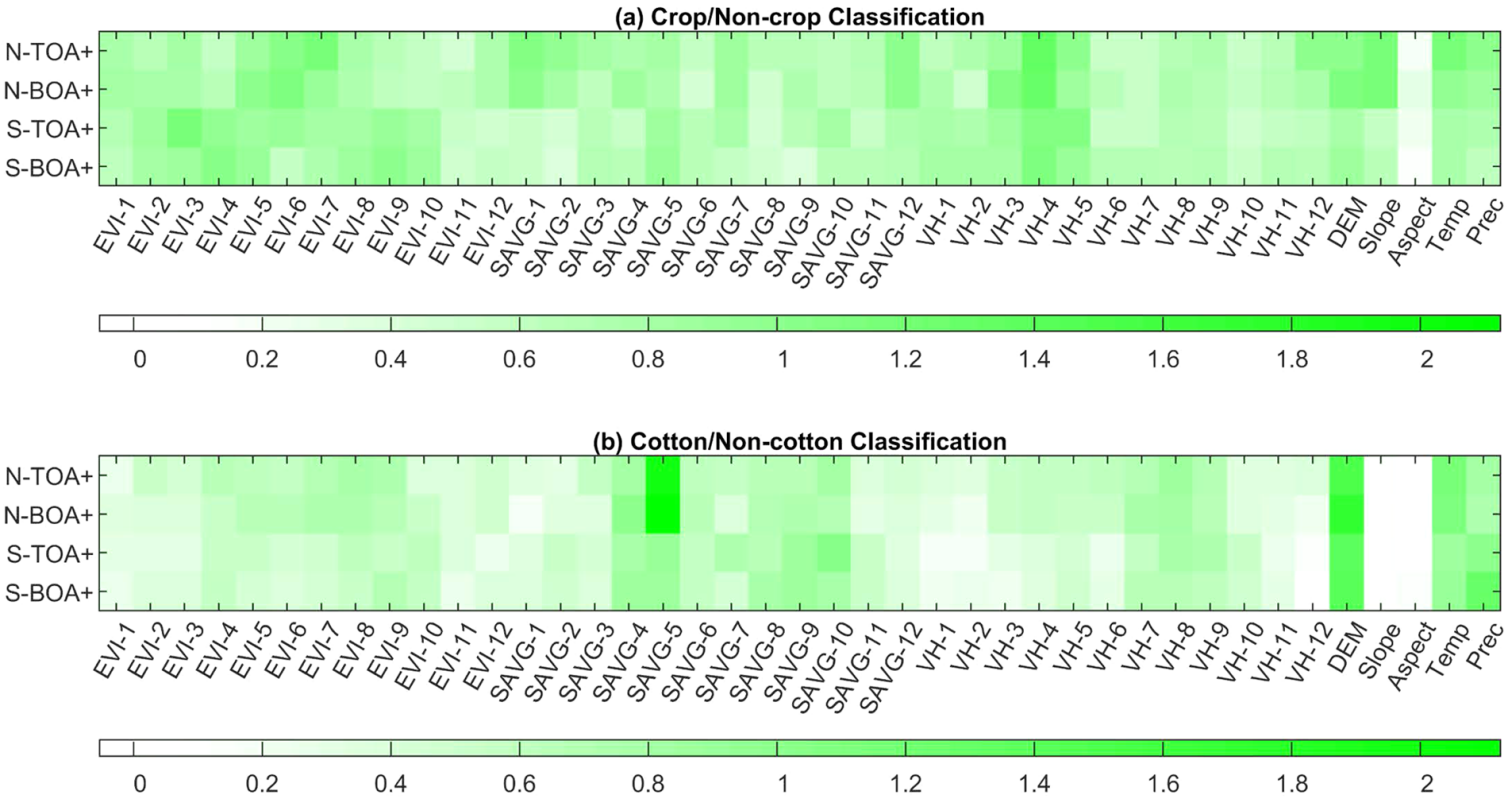
Comparison of feature importance values of feature-combinations for (**a**) Crop/Non-crop classification and (**b**) Cotton/Non-cotton classification. N and S denote Northern and Southern Xinjiang, respectively. The numbers after EVI, SAVG, and VH denote months. Temp and Prec are short for temperature and precipitation, respectively.

Clear acquaintance with the key features in crop/cotton mapping is helpful for future research. There are two common ways to derive the relative importance scores of features used in RF models. One way is based on Gini index^38^, and the other one is based on the out-of-bag (OOB) error^74,75^. Figure 8 shows the OOB error-based feature importance values (FIVs) of different classification models in this study. There were lots of discoveries in Fig. 8 which were highly consistent with Fig. 2. For example, the SAVG feature in May (SAVG-5) was one of the main features in the cotton/non-cotton classification and was more important in NX than in SX. The Aspect feature was the worst in both the crop/non-crop and cotton/non-cotton classifications, and the Slope feature was more important in NX than in SX in the crop/non-crop classification.

By contrast, FIVs of VH (Sentinel-1) were 0.75 ± 0.20 in cropland mapping and 0.45 ± 0.20 in cotton mapping; FIVs of EVI (Sentinel-2) were 0.78 ± 0.18 in cropland mapping and 0.49 ± 0.14 in cotton mapping; and FIVs of SAVG (Sentinel-2) were 0.69 ± 0.17 in cropland mapping and 0.65 ± 0.36 in cotton mapping. Results showed that the SAVG feature of Sentinel-2 contributed more than EVI (Sentinel-2) and VH (Sentinel-1) in cotton mapping (cotton/non-cotton classification). While EVI (Sentinel-2) and VH (Sentinel-1) had similar performance in cropland mapping (crop/non-crop classification), both of which had significant advantages over SAVG.

To offer more convincing explanations for the selected features, we added three additional experiments: using (1) each of vegetation indices (VIs) (EVI, NDVI (normalized difference vegetation index), NIRv (near-infrared reflectance of vegetation), and NDWI (normalized difference water index)); (2) each of SAR bands (VV and VH); and (3) each of EVI-VH (the combination of EVI and VH) and EVI-SAVG (the combination of EVI and SAVG(Blue)) as the explanatory variables to classify cotton/non-cotton using the RF classifier in the three strategies of M20, M21, and M20T21 (Fig. S1). Results showed that, first, among the optical VIs, EVI achieved higher performance than the other three VIs, particularly in the strategy of M20T21; among the SAR bands, VH achieved higher performance than VV. And thus, we chose EVI and VH as two main explanatory variables for cotton/non-cotton classification. Second, the combination of EVI-VH achieved higher and more robust accuracies than EVI and VH in terms of the three strategies of M20, M21, and M20T21 (Table S1). In particular, EVI-VH derived from Sentinel 1 and 2 achieved higher and more robust performance than EVI-SAVG considering Sentinel-2 only.

Besides, there were new findings from Fig. 8. The optical and radar features (EVI, Blue-SAVG, and VH) in the fallow period (from November to February) can make contributions to the crop/non-crop classification but gave small gains for the cotton/non-cotton classification. The reason may include that there could be apparent textural and structural differences between crop parcels and non-crop (developed land, water, desert, mountain, etc.) during the fallow periods of crops. However, it may be hard to distinguish different croplands during their fallow periods from the perspectives of texture and structure. Therefore, these variables in the fallow period can be eliminated in the cotton/non-cotton classification in the future. As the two long-term indicators, annual mean temperature (Temp) and precipitation (Prec) contributed significantly to identifying both cropland and cotton. All the three time-series features provided relatively balanced contributions, indicating their complementary advantages.

### Summary

**Fig. 9:**
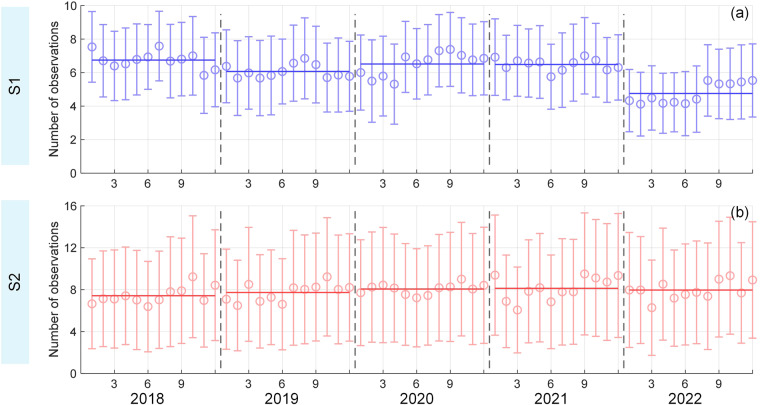
Average number of clear observations (the circles) of (**a**) S1 and (**b**) S2 across Xinjiang in each month. The error bars denote the standard deviation. A solid horizontal line indicates the average level of clear observations of all available months in a year.

Our 10-m cotton maps from 2018 to 2021 were derived from our two-step classification models with high and satisfactory accuracy and exhibited good visualization and realistic dynamics of cotton across Xinjiang. In this study, we employed the monthly interval to generate time-series features, and such an interval was proven to be feasible in weakening intra-class differences and improving inter-class separability (Fig. 2). Besides, You *et al*.^22^ reported that the monthly interval can meet the demand that almost all locations have at least one clear observation (after cloud and cloud shadow mask) in each period, but other intervals (i.e., 10-, 15-, and 20-day) cannot. Besides, the monthly composite may further weaken the influence of cotton phenological differences within the same geographical zone (Fig. 1c)^76^, thereby yielding cotton/non-cotton classification models with higher performance. In addition, the monthly composite-based classification models could be more concise and require lower cloud computing capabilities than other composite ways (i.e., 10-, 15-, and 20-day). The Sentinel 1B satellite has not worked since December 23^rd^, 2021, according to the news about the Sentinel-1 mission (https://sentinels.copernicus.eu/web/sentinel/missions/sentinel-1 (last access: June 3, 2023)). Despite the fact that Sentinel-1A was already nearing its maximum capacity, the number of effective observations per unit time of S1 was still significantly reduced, as shown in Fig. 9a. At present, enough observations (at least once a month) can still be provided by the S1 and S2 products (Fig. 9). Therefore, the proposed two-step strategy for cotton mapping can be directly transferred to 2022 in future work. However, numerous limitations and deficiencies are worth considering in future research.

First, besides the four 10-m bands (B2, B3, B4, and B8), there are other eight bands with relatively coarse resolutions (i.e., 20-m and 60-m) in S2 imagery. In order to produce the 10-m cotton maps, we only take the bands with the 10-m resolution as the basic variables. However, except for the 30-m DEM and the about 900-m WorldClim products, the GEE platform cannot provide more refined auxiliary datasets. The inconsistent resolutions of the basic and auxiliary variables probably increased the uncertainty. Besides, our classification models adopted features from multiple sensors, considering the strong independencies among different sensors. On the one hand, multiple vegetation indices and red-edge bands are often employed in the mapping of major crops^22,28,44^, indicating they may be suitable for cotton mapping in Xinjiang, but on the other hand, multiple time-series vegetation indices and bands as independent variables may lead to the Hughes phenomenon^77^. Therefore, choosing how many and which vegetation indexes as explanatory variables for cotton mapping is worth attempting in the future.

Second, earliest identifiable timing (EIT) and early-season mapping of crops are highly important for crop monitoring and yield forecasting^21,28,78^. The harvest stage of cotton in Xinjiang is mainly from October to November^13,79^. As illustrated in Fig. 2, the strong separability between cotton and non-cotton crops can be given by EVI from June to September, SAVG from April to June, and VH from June to September. And thus, the selected three features had the potential for EIT and early-season mapping of cotton in this study.

Third, some typical phenological features in the flowering and boll-opening stages may have potential for cotton mapping. However, it is hard to recognize the cotton flower by the optical imagery of Sentinel 2, mainly because there are more than ten flower colors for cotton, including white and red colors that have obvious color differences^80,81^. Particularly, (1) petal colors could change during flower development^80^; (2) there are also significant differences in color changes for different cotton varieties^80^; and (3) even on the same cotton plant, flowers of different colors coexist^82^. The boll-opening stage is another important phenological period. Most cotton has white fibre, and a small amount of cotton has colored fibre in Xinjiang^83,84^. A few recent studies have reported that the phenological feature in the boll-opening stage could be used for cotton mapping^11^ and cotton yield estimation^85–88^. Wang *et al*. (2021) proposed a white bolls index (WBI) using Sentinel 2 imagery in the boll-opening stage and performed cotton mapping in a county in Xinjiang^11^. WBI achieved the mapping performance (OA = 95% and Kappa = 0.88) that is similar to ours in this study, but it may have the following deficiencies: first, it needs the time-series data at a higher temporal resolution (5-day) to determine the time of boll-opening. In other words, it does not reduce the required data, in comparison with the monthly vegetation index used in our work. Second, it relies heavily on the optical imagery in the boll-opening period. However, it is difficult to guarantee that all cotton parcels have at least one clear observation during the boll-opening stage throughout Xinjiang due to the influence of the bad weather (clouds, mists, and rain). Third, it is hard for the approach of WBI to be transferred to the earliest identification of cotton. In particular, WBI is used to distinguish cotton and non-cotton, and hence it is essential to obtain the cropland using another technical method.

In addition, the classification models need to be improved further because the uncertainties increased when the models were transferred to adjacent years. Up to now, more and more case studies indicate that deep learning may have stronger generalization capability and transferability in crop classification than traditional machine learning^89–91^. However, deep learning models are performed on high-performance computers, and the present public platforms, including GEE, do not provide the service for deep learning. If only a deep learning strategy is used for 10-m cotton mapping in Xinjiang, it will be very expensive. The cropland area accounts for about 3% of the total land area in Xinjiang^40,55^. A compromise scheme, i.e., cropland mapping by traditional machine learning and then cotton mapping by deep learning, may be promising.

To make comparisons between the used RF models and deep learning-based models, we added an additional experiment of a spatio-temporal data-driven deep learning architecture named CNN-LSTM (a hybrid network combining convolutional neural network (CNN) and long short-term memory (LSTM))^92,93^ for the cotton/non-cotton classification (Fig. S2): first, we pre-processed and downloaded the surface reflectance data of all samples and their surrounding pixels (monthly synthesis) from GEE; second, similar to the model evaluation of RF classification performed on the GEE platform, we adopted the three strategies M20, M21, and M20T21 for the CNN-LSTM model in MATLAB 2021b (The MathWorks, Inc., Natick, MA, USA) installed on a personal computer. As shown in Fig. S3, the CNN-LSTM model achieved competitive performance in cotton/non-cotton classification for M20 and M21 but relative low performance for M20T21 under different combinations of bands (CBs) and patch sizes, compared with the proposed RF model based on multi-source data (Table 5). For M20T21, the best CNN-LSTM model was found when CBs = 9 and patch size = 5. It had an OA of 85.83%, a Kappa of 0.69, and an F1-score of cotton of 88.93%. This model was significantly worse than the proposed RF model based on multi-source data (Table 5). Considering that cotton maps in 2018, 2019, and 2021 were created using the model trained on the samples in 2020, we would prefer to choose the RF classification model instead of CNN-LSTM. Further research into the new architectures, such as the network structure considering the spatiotemporal attention mechanism, may be promising in improving the interannual transferability^94,95^.

## Usage Notes

Xinjiang is the leading and largest cotton production region in China and has accounted for over 80% and about 20% of cotton production in the whole nation and the world, respectively^5,6^. In this study, we provided the XJ_COTTON10 product^64^ (Fig. 6) from 2018 to 2021 through supervised classification using high-quality in-field samples and multi-source remote sensing data on the GEE platform. Compared with the latest published cotton mapping products^15,16^, the cotton maps of the four years in this study exhibited better spatial details and more realistic dynamics of cotton across Xinjiang (Fig. 7). In comparison with the two series of yearbooks in Xinjiang, the cotton maps gave the estimated areas with high accuracy of cotton at the county level. These novel 10-m cotton maps, which explained an average of 89.7% of the spatial variabilities in the planting areas at the county level during 2018–2020, could be helpful for high-precision cotton monitoring and policymaking across the whole province and cotton yield and production forecasting at the field parcel scale (Fig. 7).

### Supplementary information

Supplementary Material

## Data Availability

The GEE codes used to generate the cropland and cotton maps are available to the public at https://zenodo.org/record/7856467^64^.
