# Supplementary material for: The 10-m cotton maps in Xinjiang, China during 2018–2021
Source: Sci Data. 2023 Oct 10;10:688. doi: 10.1038/s41597-023-02584-3 (PMC10564865; doi:10.1038/s41597-023-02584-3)
Supplement: Supplementary file 1 — Supplementary Material [file 41597_2023_2584_MOESM1_ESM.docx]

Supplementary Material for

### The 10-m cotton maps in Xinjiang, China during 2018-2021

Xiaoyan Kang^1†^, Changping Huang^1,2†※^, Jing M Chen^3,4^, Xin Lv^5^, Jin Wang^1^, Tao Zhong^1,2^, Huihan Wang^5^, Xianglong Fan^5^, Yiru Ma^5^, Xiang Yi^5^, Ze Zhang^5※^, Lifu Zhang^1,5※^, and Qingxi Tong^1^

1. National Engineering Research Center of Satellite Remote Sensing Applications, Aerospace Information Research Institute, Chinese Academy of Sciences, Beijing 100101, China;

2. University of Chinese Academy of Sciences, Beijing 100049, China;

3. Department of Geography and Planning, University of Toronto, Toronto, ON M5S 3G3 Canada;

4. School of Geographical Sciences, Fujian Normal University, Fuzhou, China;

5. Xinjiang Production and Construction Corps Oasis Eco-Agriculture Key Laboratory, College of Agriculture, Shihezi University, Shihezi 832003, China.

Corresponding authors: Changping Huang (huangcp@aircas.ac.cn); Ze Zhang (zhangze1227@shzu.edu.cn); Lifu Zhang (zhanglf@aircas.ac.cn)


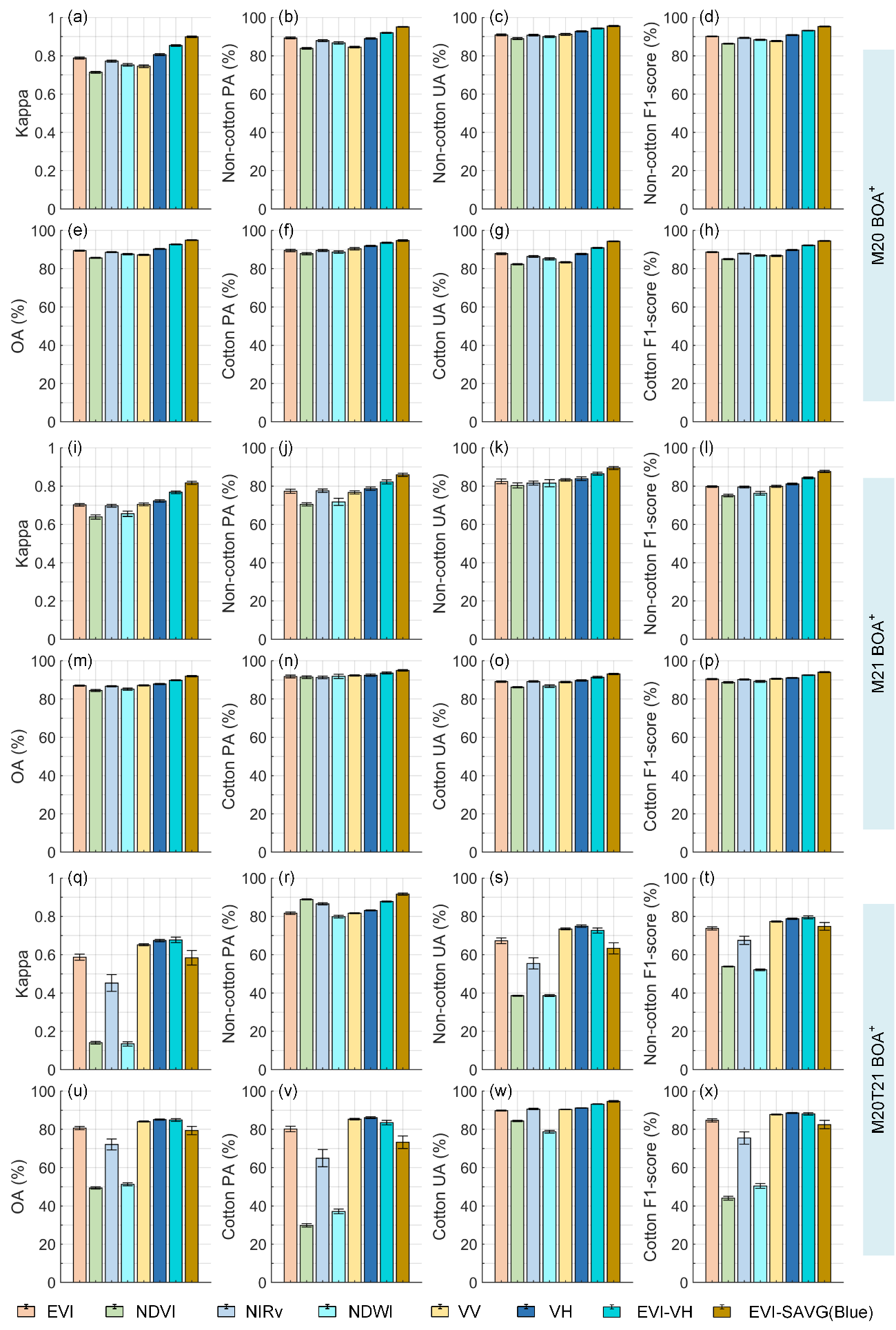


Fig. S1 Performance comparisons between Sentinel-1, Sentinel-2, and their combination in the random forest classification of cotton/non-cotton via the BOA^+^ approach. M20, M21, and M20T21 are the same to those in Table 5. Error bars show the 95% confidence intervals of the performance indicators.


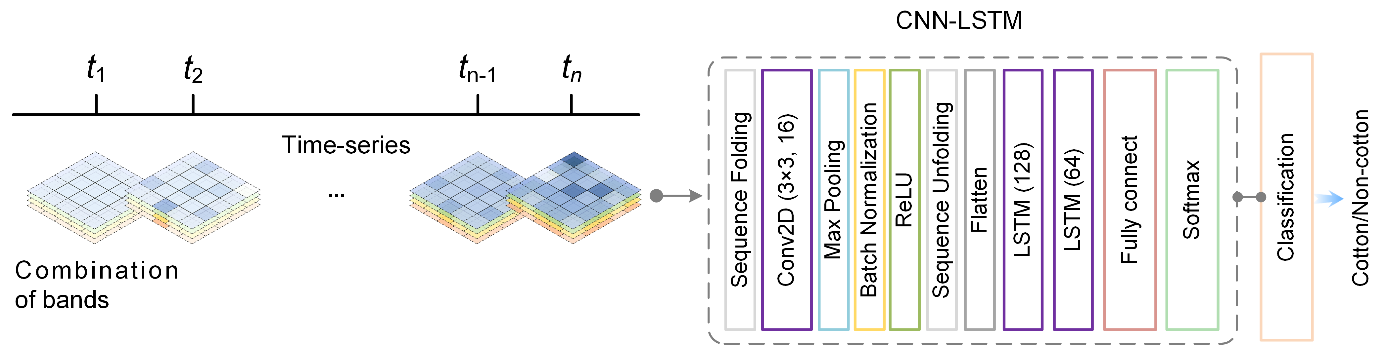


Fig. S2 The designed architecture of CNN-LSTM model for the classification of cotton/non-cotton. In this network, CNN, Conv2D, ReLU, and LSTM represent the two-dimensional convolutional neural network, the two-dimensional convolution layer, the Rectified Linear Unit layer, and the long short-term memory network, respectively. In Conv2D(m, n), there are n filters with a filter size of m; the number u of LSTM(u) is the number of hidden units.


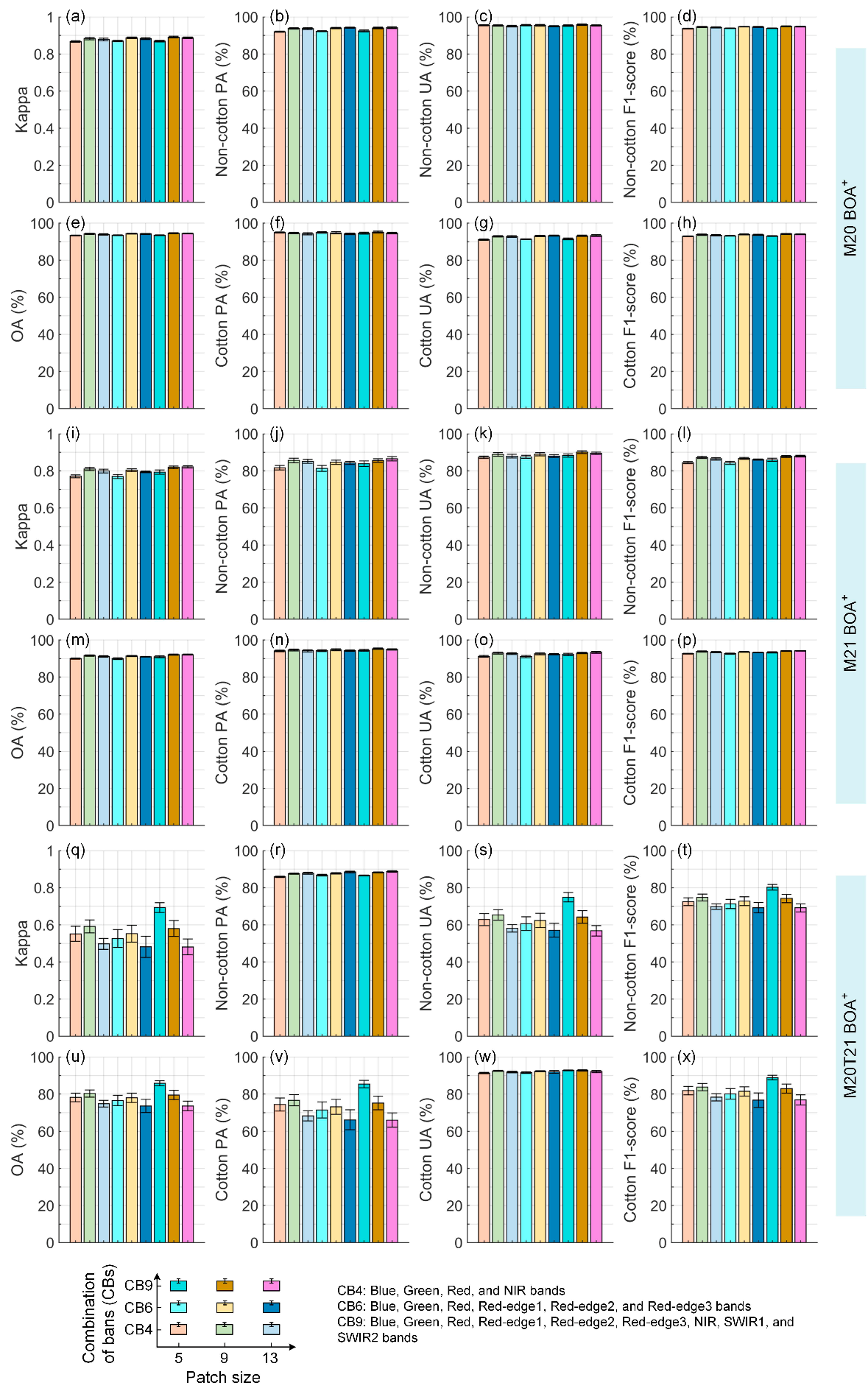


Fig. S3 Performance comparisons between different CNN-LSTM models with diverse CBs and patch sizes in the cotton/non-cotton classification using the Sentinel 2 BOA reflectance imagery. M20, M21, and M20T21 are the same to those in Table 5. Error bars show the 95% confidence intervals of the performance indicators.

**Table S1**. Average performance comparisons of EVI, VH, and their combination in cotton/non-cotton classification in the three strategies of M20, M21, and M20T21.

| Evaluation metric | EVI | VH | EVI-VH |
| --- | --- | --- | --- |
| Kappa | 0.6924 | 0.7342 | 0.7662 |
| OA (%) | 85.71 | 87.79 | 89.16 |
| Cotton F1-score (%) | 87.95 | 89.81 | 90.94 |
